# Simultaneous VENTANA IHC and RT-PCR testing of ALK status in Chinese non-small cell lung cancer patients and response to crizotinib

**DOI:** 10.1186/s12967-018-1468-9

**Published:** 2018-04-11

**Authors:** Chun-wei Xu, Wen-xian Wang, Yan-ping Chen, Yu Chen, Wei Liu, Li-hua Zhong, Fang-fang Chen, Wu Zhuang, Zheng-bo Song, Xiao-hui Chen, Yun-jian Huang, Yan-fang Guan, Xin Yi, Tang-feng Lv, Wei-feng Zhu, Jian-ping Lu, Xiao-jiang Wang, Yi Shi, Xian-dong Lin, Gang Chen, Yong Song

**Affiliations:** 1 Department of Pathology, Fujian Cancer Hospital, Fujian Medical University Cancer Hospital, No 420, Fuma Road, Fuzhou, 350014 Fujian People’s Republic of China; 2 Department of Chemotherapy, Zhejiang Cancer Hospital, Hangzhou, 310022 Zhejiang People’s Republic of China; 3 Department of Medical Oncology, Fujian Cancer Hospital, Fujian Medical University Cancer Hospital, Fuzhou, 350014 Fujian People’s Republic of China; 4 Department of Thoracic Surgery, Fujian Cancer Hospital, Fujian Medical University Cancer Hospital, Fuzhou, 350014 Fujian People’s Republic of China; 5 Geneplus-Beijing, Beijing, 102200 People’s Republic of China; 6 Department of Respiratory Medicine, Jinling Hospital, Nanjing, 210002 Jiangsu People’s Republic of China

**Keywords:** NSCLC, ALK, VENTANA IHC, RT-PCR, NGS, Crizotinib

## Abstract

**Background:**

*ALK* rearrangement-advanced NSCLC patients respond to crizotinib. *ALK* rearrangement is currently determined with RT-PCR. VENTANA IHC is a standard method to identify ALK protein overexpression in NSCLC; however, VENTANA IHC has rarely been used to determine the response to crizotinib in Chinese patients with NSCLC and ALK overexpression. To better clarify the clinical implication of VENTANA IHC to detect *ALK* rearrangements, we conducted this study to analyze VENTANA IHC and RT-PCR in a large cohort of Chinese patients with NSCLC undergoing screening for *ALK* rearrangements.

**Methods:**

A total of 1720 patients with NSCLC who had *ALK* rearrangements detected by VENTANA IHC and/or RT-PCR were included in this analysis. We compared the efficacy and survival of ALK-positive patients detected by VENTANA IHC and RT-PCR. We used NGS to identify patients in whom the two methods were inconsistent.

**Results:**

Among 1720 patients, 187 (10.87%) were shown to be ALK-positive by VENTANA IHC and/or RT-PCR, and 66 received crizotinib treatment. We identified 10.27% (172/1674) of patients as ALK-positive by the VENTANA IHC method, and 12.73% (41/322) of patients had *ALK* rearrangements by the RT-PCR method. Twenty-nine of 276 (10.51%) ALK-positive patients were simultaneously analyzed using VENTANA IHC and RT-PCR. The overall response rates were 65.90% (29/44) by VENTANA IHC and 55.88% (19/34) by RT-PCR. The disease control rates were 86.36% (38/44) by VENTANA IHC and 76.47% (26/34) by RT-PCR. In contrast, the median progression-free survival for VENTANA IHC and RT-PCR was 8.5 and 9.2 months, respectively. The VENTANA IHC and RT-PCR results obtained for 6 of 17 ALK-positive patients were inconsistent based on NGS; specifically, 4 patients had *EML4*-*ALK* fusions, 2 patients had non *EML4*-*ALK* fusions, 1 patient had a *KCL1*-*ALK* fusion, and one patient had a *FBXO36*-*ALK* fusion.

**Conclusions:**

VENTANA IHC is a reliable and rapid screening tool used in routine pathologic laboratories for the identification of suitable candidates for *ALK*-targeted therapy. VENTANA IHC has moderate sensitivity and a slightly higher association with response to therapy with ALK inhibitors, and some VENTANA IHC-positive, but RT-PCR-negative cases may benefit from crizotinib.

## Background

Lung cancer is one of the most common malignant tumors worldwide [1]. Detecting driver genes in patients with non-small cell lung cancer (NSCLC) is the new standard for clinical decision-making [2, 3]. Detecting somatic mutations within epidermal growth factor receptor (*EGFR*) and anaplastic lymphoma kinase (*ALK*) driver gene status has become a diagnostic routine for lung adenocarcinoma [4–6]. Therefore, detecting driver gene mutation status is the key to guide therapeutic decisions in clinical practice.

Crizotinib, a dual ALK–MET inhibitor, was approved by the Food and Drug Administration (FDA) in the United States for the treatment of advanced-stage NSCLC harboring an ALK rearrangement [7, 8]. One key issue in the detection of altered *ALK* is the method that best defines ALK status in a clinical setting. Currently, the diagnostic gold standard for detecting *ALK* rearrangements is break-apart fluorescence in situ hybridization (FISH), which is able to detect a large majority of *ALK* rearrangements, especially *EML4*-*ALK*, and has been clinically validated in crizotinib clinical trials [9–11]. *ALK* FISH, however, is fraught with technical and financial problems, including FISH signal instability and scoring difficulties. An alternative method for determining *ALK* rearrangements in NSCLC is a reverse transcription-polymerase chain reaction (RT-PCR). The Chinese FDA has approved the ADx *EML4*-*ALK* fusion diagnostic kit to be used for RT-PCR to detect *ALK* rearrangements; this assay has also been accepted by the Chinese Anti-cancer Association, but the requirement for fresh frozen tissue samples for extracting RNA has limited the application in clinical practice. Thus, the immunohistochemical method is a standard method to identify ALK protein overexpression in NSCLC. VENTANA immunohistochemistry (IHC) is relatively inexpensive, quick, and is performed routinely in most surgical pathology practices. VENTANA IHC was approved to detect ALK protein in pathology practice in European and some Asian countries. Currently, the response to crizotinib in NSCLC patients overexpressing ALK detected by VENTANA IHC is largely unknown. Recent developments in next-generation sequencing (NGS) of DNA and RNA have created a new opportunity for simultaneous detection of a large number of gene fusions with known and unknown partner genes and parallel detection of gene mutations [12–15]. The results of successful screening for oncogenic fusions using the NGS method have been recently reported [13].

To better clarify the clinical implication of VENTANA IHC to detect *ALK* rearrangements, we conducted this study to analyze VENTANA IHC and RT-PCR in a large cohort of screening for *ALK* rearrangements in NSCLC. Seventeen inconsistent cases with VENTANA IHC and RT-PCR underwent targeted NGS. Therefore, we investigated the response to crizotinib among Chinese NSCLC patients who overexpressed ALK as detected by VENTANA IHC, and compared the clinical value with the RT-PCR method.

## Methods

### Patients and procedures

Eligible patients were required to have pathologically-confirmed NSCLC and sufficient tissue for analysis. ALK-positivity was assessed with either VENTANA IHC or RT-PCR. Clinical and pathologic data prospectively collected for analyses included age at the time of diagnosis, gender, smoking status, stage, histology, specimen type, and *EGFR* status according to the new International Association for the Study of Lung Cancer, American Thoracic Society, and European Respiratory Society multidisciplinary classification. A subset of patients received crizotinib treatment (250 mg twice daily) and had clinical data available on the outcome. Imaging data were independently reviewed by authors to evaluate the treatment responses according to the Response Evaluation Criteria in Solid Tumors (RECIST; version 1.1). Progression-free survival (PFS) was calculated from the date of initiating targeted drug treatment to radiologic or clinical observation of disease progression. This study was approved by the Ethics Committee of Fujian Cancer Hospital (Fujian Medical University Cancer Hospital, Fuzhou Fujian, China) and written informed consent was obtained from each participant before the initiation of any study-related procedure.

### VENTANA immunohistochemistry

Immunohistochemistry was carried out on a fully automated VENTANA Benchmark XT stainer (VENTANA Medical Systems; Roche Group, Tucson, AZ, USA) using the pre-diluted VENTANA anti-ALK (D5F3) rabbit monoclonal primary antibody, together with the Optiview DAB IHC detection and Optiview amplification kits (VENTANA Medical Systems; Roche Group, Tucson, AZ, USA). Each case was also stained with a matched rabbit monoclonal negative control immunoglobulin antibody. A binary scoring system was adopted for evaluating the staining results. The presence of strong granular cytoplasmic staining in tumor cells (any percentage of positive tumor cells) was deemed to be ALK-positive, while absence of strong granular cytoplasmic staining in tumor cells was deemed to be ALK-negative.

### RNA preparation

The RNA was extracted from lung tumors per standard protocols (RNeasy Mini Kit; Qiagen, Hilden, Germany or AmoyDx RNA Kit; Amoy Diagnostics Co., Xiamen, China). This extraction method was optimized by the manufacturer to reverse formaldehyde modification without further RNA degradation and has been shown to be an efficient method to obtain RNA of sufficient quantity for PCR amplification in our laboratory.

### *ALK* rearrangement detection

RT-PCR was used to detect the *ALK* rearrangement. The *ALK* rearrangement mRNA was detected using the AmoyDx *EML4*-*ALK* Fusion Gene Diagnostic Kit (Cat No. ADx-FF04), which is designed to detect 21 types of known *ALK* rearrangements, including E6;A19, E6;A20, E6ins33;A20, E6;ins18A20, E13;A20, E13;ins69A20, E20;A20, E20;ins18A20, E14ins11;del49A20, E14;del14A20, E14;del38A20, E15del60;del71A20, E2;A20, E2;ins117A20, E3;ins53A20, E17;ins30A20, E17ins61;ins34A20, E17ins65;A20, E17;ins68A20, E17del58;ins39A20, and E18;A20. In brief, the mRNA extracted from the previous step was reverse-transcribed to cDNA at 42 °C, followed by PCR amplification. The PCR condition of the cDNA was as follows: initial denaturation at 95 °C for 5 min, followed by 95 °C for 25 s, 64 °C for 20 s, 72 °C for 20 s to ensure the specificity; and up to 31 cycles at 93 °C for 25 s, 60 °C for 35 s, and 72 °C for 20 s. The details are described in our previous studies [16, 17].

### Targeted next-generation sequencing

For 17 patients in whom the two methods were inconsistent, targeted region capture combined with NGS was performed. Genomic DNA sequencing libraries were prepared using the protocols recommended by the Illumina TruSeq DNA Library Preparation Kit (Illumina, San Diego, CA, USA). For samples close to the minimum input requirement, additional pre-capture PCR cycles were performed to generate sufficient PCR product for hybridization. The libraries were hybridized to custom-designed probes (Integrated DNA Technology, Coralville, IA, USA), including all exons of 170 genes and selected introns of ALK, RET, and ROS1 for the detection of genomic rearrangements. DNA sequencing was performed on a HiSeq3000 sequencing system (Illumina, San Diego, CA, USA) with 2 × 75 bp paired-end reads. The reads were aligned to the human genome build GRCh37 using a Burrows–Wheeler aligner (BWA). Somatic single nucleotide variant (sSNV) and indel calls were generated using MuTect and GATK, respectively. Somatic copy number alterations were identified with CONTRA. Genomic rearrangements were identified by the software developed in-house analyzing chimeric read pairs.

### Statistical analysis

A Chi square or Fisher’s exact test was used to analyze correlations between ALK status and the clinicopathologic factors. The response rate among subgroups and survival were described with Kaplan–Meier methodology and the log-rank test was used to compare survival among subgroups. Statistical analysis was performed using SPSS (version 19.0 software; IBM, Armonk, NY, USA). All p values were two-sided, and a p < 0.05 was considered statistically significant.

## Results

### Patient characteristics

**Fig. 1:**
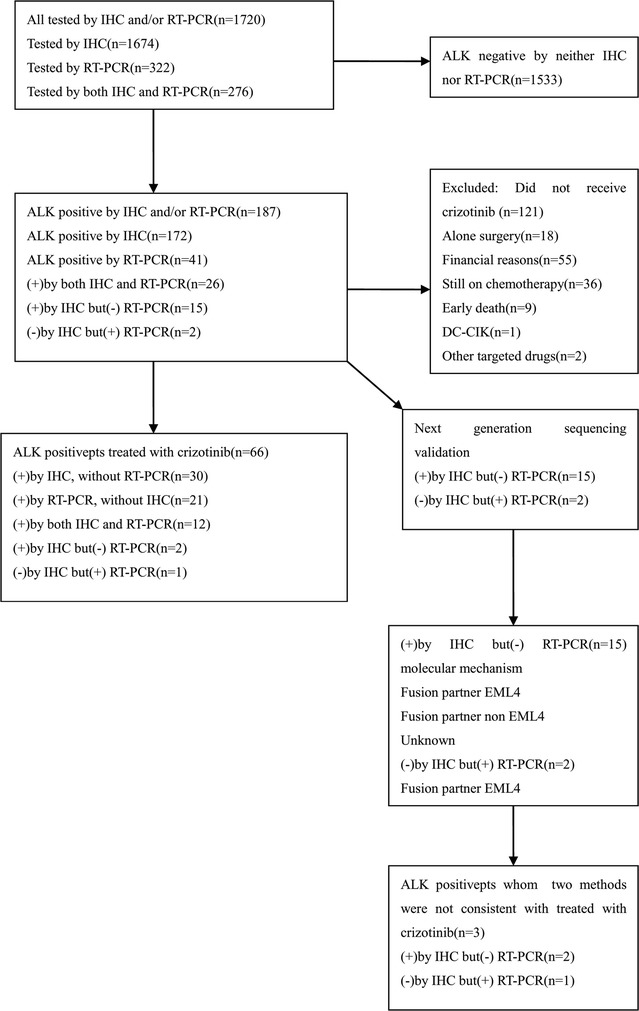
Flow chart of the study design

From July 2013 to May 2016 a total of 1720 patients were enrolled in this study. Among the patients, 187 (10.87%) were identified as ALK-positive and 66 received oral crizotinib. The flow chart of the study design is shown in Fig. 1. The baseline clinical characteristics are shown in Table 1. The patients harboring *ALK* rearrangements had the following characteristics: younger (median age 51 vs. 69 years, p < 0.001); never-smokers (p < 0.001), IIIb–IV stage disease (p < 0.001); and wild-type EGFR (p < 0.001). There was no statistical differences based on gender, histology, and specimen type (Table 1).

**Table 1:** The clinical characteristics of patients

Clinical characteristics	Patients (n = 1720)			Crizotinib treated (n = 66)
	ALK positive (n = 187)	ALK negative (n = 1533)	p value^a^	
Age (years), median (range)	51 (22–84)	69 (20–92)	< 0.001	54 (33–71)
Gender			0.646	
Male	114	961		42
Female	73	572		24
Smoking status			< 0.001	
Yes	13	532		4
No	174	1001		62
Stage			< 0.001	
I–IIIa	16	498		0
IIIb–IV	171	1035		66
Histology			0.121	
Adenocarcinoma	157	1213		65
Non-adenocarcinoma	30	320		1
Specimen type			0.375	
Fine needle aspirate	123	928		66
Surgical specimens	56	524		0
Cytology specimens	8	81		0
*EGFR* status			< 0.001	
Wild type	119	472		50
Mutation	13	532		0
Unknown	55	529		16

^a^ The *p* value was used to compare the clinical characteristics between *ALK* fusion positive and negative patient

### VENTANA IHC and RT-PCR analysis

**Fig. 2:**
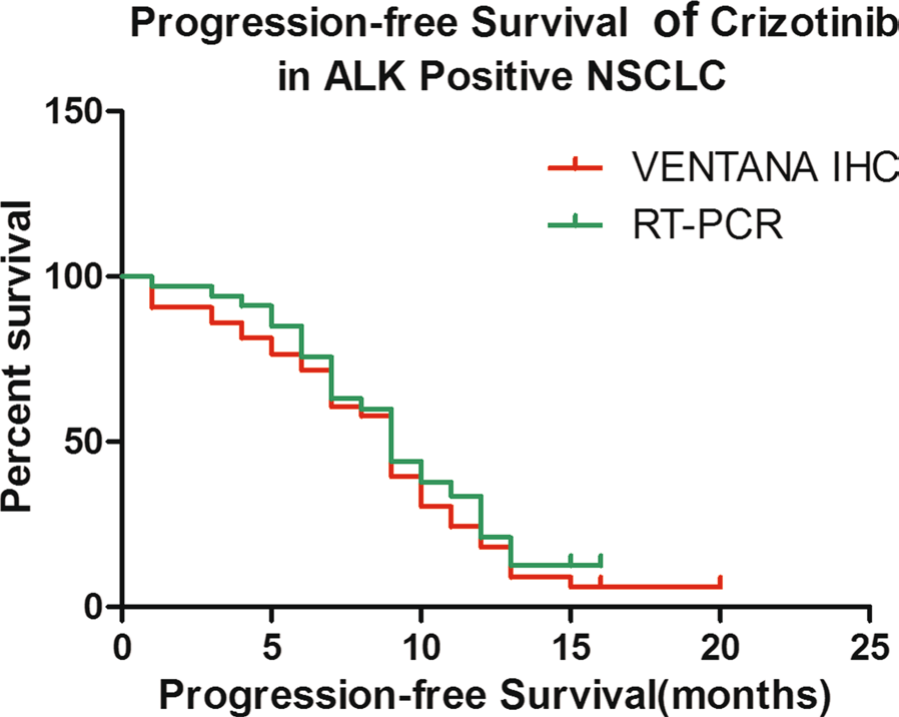
Comparison of the PFS curve in ALK-positive advanced NSCLC patients detected by VENTANA IHC or RT-PCR, and treated with crizotinib (8.5 vs. 9.2 months, p = 0.630)

Among the 1720 specimens tested with VENTANA IHC and/or a RT-PCR assay, 1674 cases were analyzed using VENTANA IHC and 322 cases were analyzed using RT-PCR, including 276 cases simultaneously analyzed by VENTANA IHC and RT-PCR. We showed that 10.27% (172/1674) of the patients were ALK-positive by the VENTANA IHC method, 12.73% (41/322) of the patients had *ALK* rearrangements by the RT-PCR method, and 26 cases of the patients were positive by VENTANA IHC and RT-PCR. The response was evaluated in the 66 ALK-positive advanced NSCLC patients who received crizotinib therapy detected by VENTANA IHC and/or RT-PCR. Among the 66 patients, 1 had a complete response (CR), 38 had a partial response (PR), and 14 had stable disease (SD). Thus, the objective response rate (ORR) was 59.09% (95% confidence interval [CI] 46.1–72.4) and the disease control rate (DCR) was 80.30% (95% CI 71.7–90.2). Subgroup analysis based on VENTANA IHC revealed that 29 achieved an objective response (OR) and 9 had SD. Thus, the ORR was 65.90% (95% CI 45.3–76.2) and the DCR was 86.36% (95% CI 74.3–93.9). In contrast, RT-PCR 19 achieved an OR and 7 had SD. Thus, the ORR was 55.88% (95% CI 31.2–70.6) and the DCR was 76.47% (95% CI 63.4–85.2). Survival analyses were performed in all 66 patients with a median duration of follow-up of 34 months (95% CI 24.74–45.58). Seventeen of 66 patients (25.76%) were still on ALK-TKIs and 26 (39.39%) were alive at the last follow-up date (31 December 2016). The median PFS was 9.0 months (95% CI 8.04–9.96), while the median overall survival (OS) has not been reached. Based on subgroup analysis, the median PFS of VENTANA IHC was 8.5 months (95% CI 7.18–10.00) vs. 9.2 months (95% CI 7.88–10.51; Fig. 2; p = 0.630) for RT-PCR.

### NGS validation of VENTANA IHC and RT-PCR inconsistent cases

**Fig. 3:**
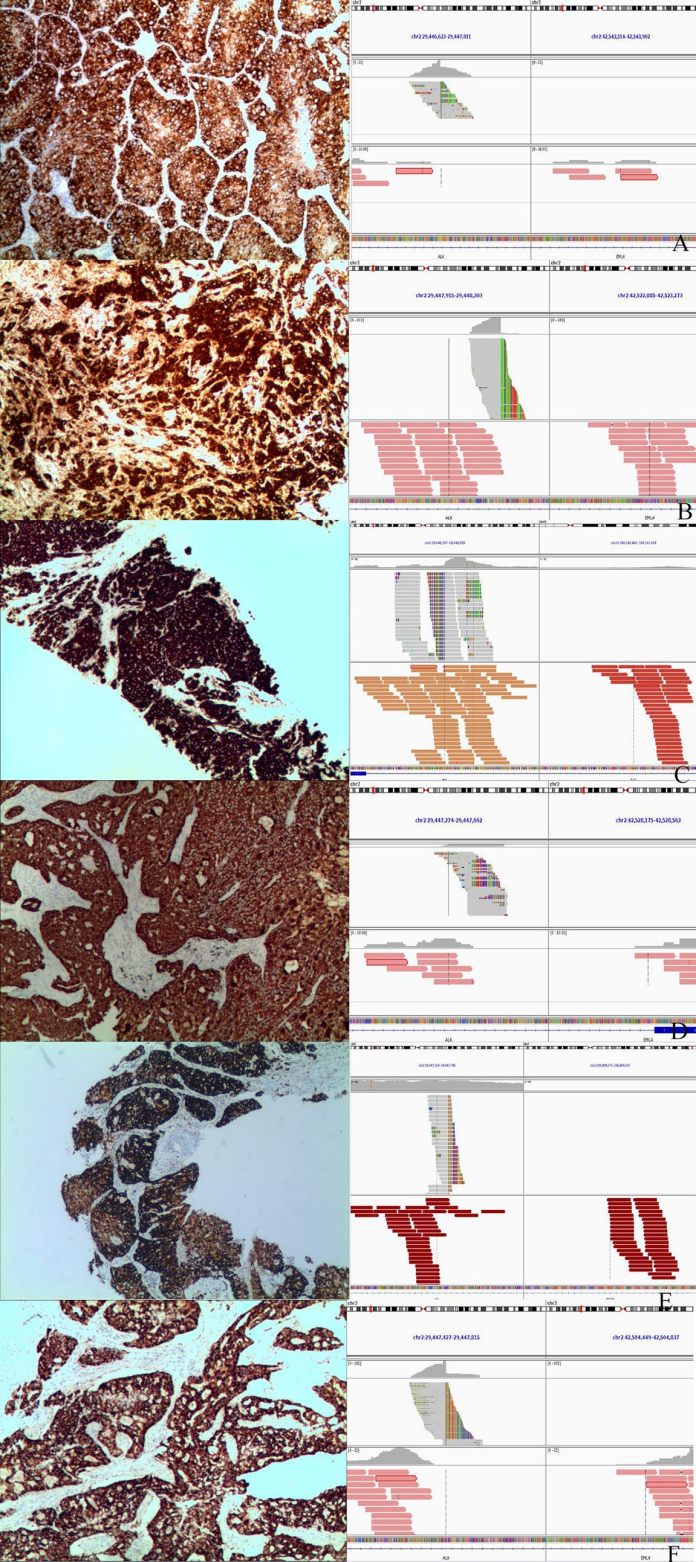
Pathologic and genetic features of six patients with VENTANA IHC positive RT-PCR negative NSCLC. By column: Positive of ALK expression with original magnification ×200 by VENTANA IHC, targeted next-generation sequencing of *ALK* gene demonstrates the BWA of the *ALK* exon 20 region around the transcription breakpoint (**A** case 2, **B** case 5, **C** case 6, **D** case 9, **E** case 12, and **F** case 15)

Seventeen of 187 cases of ALK-positive patients were inconsistent by VENTANA IHC and RT-PCR, including 15 patients who were VENTANA IHC-positive and RT-PCR negative, and 2 patients who were VENTANA IHC-negative and RT-PCR positive. Among six cases evaluated with NGS, four had *EML4*-*ALK* fusions, including E6;A20, E13;A20, E14; A20, and E18; A20 (Fig. 3, Table 2). Of the four cases with *EML4*-*ALK* fusions, 1 had *PTPN11* p.G503V and *NF1* p.Q2492*, 1 had *KRAS* p.G12C and *MSH2* p.Q629R, 1 had *TP53* p.Y205C, and 1 had *TP53* p.I162F and *EGFR* p.L858R. Two cases had non *EML4*-*ALK* fusions; 1 had a *KCL1*-*ALK* fusion (accompanied *BRCA1* p.E733Q) and the other had a *FBXO36*-*ALK* fusion (*NF1* p.A2437S; Table 2, Fig. 3). Among 17 cases, the response was evaluated in the 3 patients with ALK-positive advanced NSCLC who received crizotinib therapy; 2 patients were failed by NGS (case 3: PFS, 7.4 months and OS, 22.5 months; case 17: PFS, 1.0 month and OS, 8.4 months), 1 patient was successfully evaluated by NGS (case 12: *FBXO36*-*ALK* PFS, 21.2 months and OS, > 46.7 months).

**Table 2:** Clinicopathologic details of 15 patients with NSCLC with VENTANA IHC positive and RT-PCR negative and 2 patients with NSCLC with VENTANA IHC negative and RT-PCR positive

Case no.	Sex/age (years)	Smoking status	Sample type	Histology	ALK VENTANA IHC	ALK RT-PCR	EGFR RT-PCR	NGS partner gene	NGS variant	NGS other mutations
1	F/42	No	FNA	ADC	Pos	Neg	WT	Failed	Failed	Failed
2	M/50	Yes	SS	ADC	Pos	Neg	WT	EML4	18	PTPN11 p.G503 V NF1 p.Q2492*
3	M/53	No	FNA	ADC	Pos	Neg	WT	Failed	Failed	Failed
4	M/67	Yes	FNA	SCC	Pos	Neg	WT	Failed	Failed	Failed
5	M/72	No	SS	PSC	Pos	Neg	WT	EML4	13	KRAS p.G12C MSH2 p.Q629R
6	M/45	No	FNA	ADC	Pos	Neg	WT	KCL1	–	BRCA1 p.E733Q
7	M/45	No	FNA	ADC	Pos	Neg	L858R	Failed	Failed	Failed
8	M/51	No	FNA	ADC	Pos	Neg	WT	Failed	Failed	Failed
9	M/62	No	SS	ADC	Pos	Neg	WT	EML4	14	TP53 p.Y205C
10	F/44	No	FNA	ADC	Pos	Neg	L858R	Failed	Failed	Failed
11	F/58	No	FNA	ADC	Pos	Neg	WT	Failed	Failed	Failed
12	M/68	Yes	FNA	ADC	Pos	Neg	WT	FBXO36	–	NF1 p.A2437S
13	M/71	Yes	FNA	ADC	Pos	Neg	WT	Failed	Failed	Failed
14	M/53	No	FNA	ADC	Pos	Neg	WT	Failed	Failed	Failed
15	F/63	No	SS	ADC	Pos	Neg	L858R	EML4	6	TP53 p.I162F EGFR p.L858R
16	F/47	No	FNA	ADC	Neg	Pos	WT	Failed	Failed	Failed
17	M/54	Yes	FNA	ADC	Neg	Pos	WT	Failed	Failed	Failed

*IHC* immunohistochemistry, *NGS* next-generation sequencing, *F* female, *M* male, *FNA* fine needle aspirate, *SS* surgical specimens, *ADC* adenocarcinoma, *SCC* squamous cell carcinoma, *PSC* pulmonary sarcomatoid carcinoma, *Pos* positive, *Neg* negative, *WT* wild type, *NGS* next-generation sequencing, *Failed* poor quality of DNA or no tissues

## Discussion

*ALK* rearrangements in patients with NSCLC are highly sensitive to crizotinib treatment [18]. As first-line treatment, crizotinib has an ORR of 74% and the median PFS is 10.9 months [6]. Therefore, identification of appropriate patients with reliable detection methods is important for targeted therapy. Previous studies have demonstrated that VENTANA IHC is a highly sensitive and specific assay for detection of ALK gene status, and is a feasible alternative to the *ALK* FISH assay [19–21]. RT-PCR is an alternative method that is rapid and convenient to perform [22]; however, the requirement of fresh frozen tissue samples for extracting RNA by the RT-PCR assay has limited its application in clinical practice. In the current study, we compared VENTANA IHC with the RT-PCR assay to detect *ALK* rearrangements and report for the first time the response and survival of crizotinib for Chinese patients with *EML4*-*ALK* positive advanced NSCLC detected by VENTANA IHC and RT-PCR. In the enrolled 1720 patients, we identified 10.27% (172/1674) patients had ALK positive by VENTANA IHC method, 12.73% (41/322) patients had *ALK* rearrangements by RT-PCR method, and 9.42% (26/276) patients were both positive by VENTANA IHC and RT-PCR. It has been reported that *ALK* rearrangements range from 2 to 7% among unselected Caucasian NSCLC patients [23, 24]. The frequency has been reported to be as high as 5–10% and is higher in the Asian population [25–27]. The frequency of detection by RT-PCR in the current study was higher than VENTNANA IHC. Rosell et al. [28] showed that RT-PCR detected more cases with the *EML4*-*ALK* fusion gene (12.5%) than IHC (6.7%) among 200 NSCLC patients, and based on routine examination by the two techniques, the *EML4*-*ALK* rearrangements can be detected more frequently by RT-PCR. The VENTANA IHC assay is performed routinely in most surgical pathology practices and IHC has been demonstrated as a reliable pre-screening test for detecting lung cancer in clinical practice. In addition, we observed an ORR of 59.09%, DCR of 80.30%, and median PFS of 9.0 months in 66 ALK-positive patients. The ORR was 65.90% and the DCR was 86.36% in the 44 patients in whom an *ALK* translocation was confirmed by VETNANA IHC. The ORR was 55.88% and the DCR was 76.47% by RT-PCR in 34 ALK-positive patients. The median PFS of VENTANA IHC and RT-PCR was 8.5 and 9.2 months, respectively (p = 0.630). Although our study included first-, second-, and later-line crizotinib therapy, the median PFS was within the range of 7.7–10.9 months, as reported in relevant clinical trials [6, 10, 29]. Interestingly, we also observed responses to crizotinib in two patients who had positive ALK fusion by VENTANA IHC, but not by RT-PCR. Another case which was ALK-positive by RT-PCR, but negative by VENTANA IHC, did not show a good response to crizotinib treatment. Therefore, VENTANA IHC is a rapid and relatively inexpensive method for diagnosing *ALK*-rearranged NSCLC. RT-PCR may be highly sensitive, but the specificity of RT-PCR as a screening tool is likely to be extremely high and low abundance results accompanied other genes may occur with this highly sensitive technique [28]. The application of VENTANA IHC is more moderate than RT-PCR as a screening method to detect ALK status in clinical practice and patients could benefit from crizotinib more so than RT-PCR.

In the current study, there were six patients with ALK VENTANA IHC-positive and RT-PCR-negative, demonstrating *ALK* rearrangement revealed by NGS. Another 6 patients who had primary resistance to crizotinib therapy were also analyzed by NGS. Our results suggest that 4 patients were identified to have *EML4*-*ALK* variants, including E6; A20, E13; A20, E14; A20, and E18; A20. Two patients were identified to have non-*EML4*-*ALK* fusions; one patient with a *KCL1*-*ALK* fusion, which had been previously reported in the literature [30], and one patient with a *FBXO36*-*ALK* fusion who received crizotinib therapy with a PFS of 21.2 months and an OS of > 46.7 months. NGS revealed a new ALK partner gene, *FBXO36*, which is the first report in NSCLC worldwide, and it has good response to crizotinib. Currently, developments in NGS have created a new method for the simultaneous detection of a large number of gene fusions with known and unknown genes and gene mutations [12, 14, 15]. Pekar-Zlotin et al. [12] reported a 42.9% sensitivity and 97.7% specificity for *ALK* FISH when compared to NGS DNA-based platform for the detection of *ALK* gene rearrangements. Dacic et al. [31] also demonstrated significant concordance between IHC and NGS in cases discordant between NGS and FISH. VENTANA IHC detects ALK expression for *ALK* fusion genes independent of variant and fusion partners. Therefore, the VENTANA IHC method is highly recommended in routine pathologic diagnosis.

Although our results are significant, we recognize that there are limitations to the study. First, a major limitation was the retrospective design. Second, because of insufficient samples or DNA, we did not assess all tissues by VENTANA IHC and RT-PCR.

## Conclusions

Our study suggests that VENTANA IHC may be a reliable and initial screening approach for assessment of *ALK* rearrangements in patients with NSCLC. In addition to RT-PCR, VENTNANA IHC also identified patients who responded to crizotinib and are RT-PCR negative. NGS as a technique for detecting *ALK* gene fusions and primary resistance to ALK TKI warrant further study.

## Abbreviations

ALK: anaplastic lymphoma kinase
NSCLC: non-small cell lung cancer
PFS: progression-free survival
OS: overall survival
PR: complete response
CR: complete response
SD: stable disease
OR: objective response
DCR: disease control rate
ORR: objective response rate
